# BK Virus and Transplantation

**DOI:** 10.3390/v13050733

**Published:** 2021-04-22

**Authors:** Carla Prezioso, Valeria Pietropaolo

**Affiliations:** 1 Department of Public Health and Infectious Diseases, “Sapienza” University of Rome, 00185 Rome, Italy; carla.prezioso@uniroma1.it; 2 Microbiology of Chronic Neuro-Degenerative Pathologies, IRCSS San Raffaele Pisana, 00163 Rome, Italy

As guest editors, we are pleased to present this Special Issue on BK virus (BKV) and transplantation with the intention of exploring some aspects related to BKV-associated diseases in transplant recipients, since they are still unclear. It is well known that BKV-associated diseases in transplant recipients are an emerging issue and that kidney transplant recipients (KTRs) comprise the patient population that most frequently experiences complications of BKV reactivation, that can be linked to a BKV-associated nephropathy (BKVN) with a significant risk of allograft loss. Yet, there are still numerous unanswered questions about the mechanism of viral persistence and the conditions that lead to viral reactivation upon immunosuppression; the role that innate immune mediators play in controlling BKV infection; the role of viral subtypes and subgroups in the development of clinical syndromes and nephropathy; the study of new antiviral agents for BKV infection, since the mainstay of managing reactivation is reduction of immunosuppression; the development of immune-based therapies to combat BKV and BKV-associated complications and the development of improved diagnostic tools and clinical management strategies, useful for tracing infection trails in epidemiologic investigations.

The breadth of articles and reviews in this issue provides valuable insight on BKVN and allograft rejection, on recent advances in molecular diagnosis and immunological therapy for BKVN patients and on the development of new biomarkers for BKVN diagnosis.

The review by Shen et al. [1] in this issue underlines that BKVN and allograft rejection are two closely associated diseases on opposite ends of the immune scale in kidney transplant recipients and that the principle of balancing the immune system remains the mainstay of therapeutic strategy. The article discusses the delicate evaluation needed to optimize immunosuppression and reviews recent advances in molecular diagnosis and immunological therapy for BKVN patients, focusing on new biomarkers for BKVN diagnosis under development, such as the measurement of virus-specific T cell level, and on the development of cellular therapy that may prevent, or even cure, BKVN.

Additionally, numerous risk factors associated with BKV infection must be taken into account in BKAN management. The review by Burek Kamenaric et al. [2] summarizes current knowledge on the association between human leukocyte antigens (HLAs), killer cell immunoglobulin-like receptors (KIR) alleles and BKV infection. Assessment of risk involving HLA ligands and KIR genotyping of recipients in the pre-transplant or early post-transplant period might be useful in prevention or early action to reduce morbidity and progression to nephropathy.

The importance of the neutralizing antibody response in controlling BKV replication in KTRs has been highlighted by McIlroy et al. [3], who investigated the relationship between neutralization escape and persistent high-level BK polyomavirus replication after kidney transplant, determining viral capsid protein 1 (VP1) sequences in longitudinal samples from KTRs with persistent high-level viruria compared to patients who suppressed viruria. Interesting and promising results were obtained in this study. In fact, persistent high-level BK polyomavirus replication in KTRs is associated with the accumulation of VP1 mutations that can confer resistance to neutralization, implying that future BKV therapies involving monoclonal antibodies may be more effective when used as preventive or pre-emptive, rather than curative, strategies.

Reactivation of BKV infection highlights the importance of immune system components in controlling viral reactivation. The role of antiviral cytokines in infection control in particular are poorly understood. In the paper of Fiore et al. [4], the efficacy of interferons (IFNs) alpha, lambda and gamma with regard to BKV multiplication in Vero cells was investigated, analyzing the possible antiviral role of indoleamine 2,3-dioxygenase (IDO, an enzyme involved in tryptophan catabolism) secreted by IFN-gamma-stimulated cells. Results indicated that IDO has a specific role in anti-BKV activity. A better understanding of the action mechanism of these IFN-gamma-induced antiviral proteins might facilitate the development of novel therapeutic strategies. These considerations emphasize the importance of developing an in vivo model (such as an IDO/mouse) in which to study the true impact of IDO on BKV.

Interestingly, the use of inhibitors of the mechanistic target of rapamycin (mTORi) could also be favorable in case of viral infection. Reactivations of BKV and human cytomegalovirus (HCMV) are dependent on the mTOR pathway for replication. Cherneha and colleagues [5] investigated the association of viral replication with mTOR activity in peripheral lymphocytes of renal transplant recipients, using a flow cytometry-based assay for the measurement of Thr389 p70S6k phosphorylation, a surrogate marker of the mTOR pathway. Results evidenced that episodes of BKV replication were significantly associated with increased p70S6k phosphorylation in CD4+ T lymphocytes and CD19+ B lymphocytes. If infections with viruses like BKV and HCMV lead to a measurable increase in p70S6k phosphorylation of peripheral lymphocyte populations, this method could be of use in transplant recipient monitoring, especially in respect to the difficult and invasive diagnosis of BKVN by kidney biopsy.

Although the microRNAs (miRNAs) bkv-miR-B1-3p and bkv-miR-B1-5p are produced during the viral cycle, their putative value as markers of viral replication has yet to be established. In KTRs, the clinical relevance of the changes over time in BKV miRNA levels has not been determined. In this scenario, the paper of Demey et al. [6] analyzed, in a retrospective study, urine samples and plasma samples collected from KTRs during the first year post-transplantation. Seven of the 14 KTRs with a sustained BKV infection showed a progressive reduction in DNA load and then a rapid disappearance of miRNAs. These results are encouraging to open up the use of bkv-miR-B1-3p and bkv-miR-B1-5p levels in the urine as valuable markers for viral replication monitoring and might help physicians to avoid an excessive reduction in the immunosuppressive regimen.

Emerging evidence indicates that reactivation of BKV in the kidney and urothelial tract of KTRs may be associated with cancer in these sites. In this Special Issue, three papers focus on this matter. The first is the brief report by Borgogna et al. [7], who, in a retrospective study, analyzed 10 clear cell renal cell carcinomas and five urinary bladder carcinomas from 15 KTRs for the presence of BKV infection through immunohistochemistry and fluorescent in situ hybridization (FISH) and the results obtained further strengthen the association between BKV reactivation and cancer development in KTRs, especially bladder carcinoma.

In the paper of Klufah et al. [8], by the retrospective use of a large single-institution database, the relationship of BKV-positive urine cytology specimens (UCSs) with urothelial cell carcinoma (UCC) was evaluated. UCSs positive for decoy cells were tested for the presence of BKV by polymerase chain reaction (PCR) and immunohistochemistry (IHC) in urine sediments and formalin-fixed paraffin-embedded (FFPE) tissue samples of UCC. IHC identified BKV positivity in the urine samples of non-UCC and UCC patients, while no BKV positivity was found in FFPE tissues of primary UCCs and metastases. In addition, BKV PCR results revealed the presence of BKV DNA in the urine of the UCC cases, yet none in the UCC tissue itself. These data indicate that BKV reactivation is not restricted to immunosuppression but also found in the urine of patients diagnosed with urothelial cell carcinoma and patients without any history of transplantation, malignancy or chronic diseases.

Additionally, Li and colleagues [9] aimed to verify the association between BKV infection and urinary tract cancers (UTCs), analyzing the risk of UTC among patients with and without biopsy-proven BKVN in a 20-year cohort. Results demonstrated that BKVN patients have greater allograft losses, higher incidence, a lower cancer-free survival rate and an earlier onset with a higher relative risk of developing UTC compared to non-BKVN patients.

In this issue, the quasi-species topic was also addressed. Signorini et al. [10] investigated, in a prospective observational study, the replication pattern and the genomic characterization of BKV, JC polyomavirus and Merkel cell polyomavirus infections in KTx. Results showed that after kidney transplant, the human polyomavirus (HPyV) genome remains stable over time with no emergence of quasi-species. HPyV strains isolated in donor/recipient pairs are mostly identical, suggesting that viruses detected in the recipient may be transmitted by the allograft. 

Finally, an important contribute to the issue was made by the paper of Zanotto et al. [11], that describes the occurrence of BKVN in the Turin Transplant Center (Piemonte region, northwestern Italy) over a period of five years and the outcome of these cases took into consideration the histological evaluation and the modifications in immunosuppressive treatment, supporting the relevant role played by immunomodulation as a therapeutic strategy.

In summary, each of the research articles and the reviews in this Special Issue tackles fundamental aspects related to BKV-associated diseases in transplant recipients, laying out the state of our knowledge to date on BKV and transplantation and outlining the many open questions that remain. 

## Data Availability

Not applicable.
